# Association of Molecular Biomarker Heterogeneity With Treatment Pattern and Disease Outcomes in Multifocal or Multicentric Breast Cancer

**DOI:** 10.3389/fonc.2022.833093

**Published:** 2022-06-23

**Authors:** Shuai Li, Jiayi Wu, Ou Huang, Jianrong He, Weiguo Chen, Yafen Li, Xiaosong Chen, Kunwei Shen

**Affiliations:** Department of General Surgery, Comprehensive Breast Health Center, Ruijin Hospital, Shanghai Jiao Tong University School of Medicine, Shanghai, China

**Keywords:** biomarkers, breast neoplasms, intertumoral heterogeneity, multifocal, multicentric, prognosis

## Abstract

**Purpose:**

This study aimed to evaluate the rates of estrogen receptor (ER), progesterone receptor (PR), human epidermal growth factor receptor 2 (HER2), and Ki67 heterogeneity in multifocal or multicentric breast cancer (MMBC) and its association with treatment pattern and disease outcomes.

**Methods:**

MMBC patients with ER, PR, HER2, and Ki67 results for each tumor focus were retrospectively analyzed using Kappa test and categorized into the homogeneous group (Homo group) and the heterogeneous group (Hetero group). Chi-square tests were performed to compare the clinical features and treatment options between the groups. Disease-free survival (DFS) and overall survival (OS) rates were estimated from Kaplan–Meier curves and compared between two groups.

**Results:**

A total of 387 patients were included, and 93 (24.0%) were classified into the Hetero group. Adjuvant endocrine therapy was more frequently assigned for patients in the Hetero group than in the Homo group (84.9% vs. 71.7%, *p* = 0.046). There was no difference in terms of adjuvant anti-HER2 therapy (28.3% vs. 19.6%, *p* = 0.196) and chemotherapy (69.9% vs. 69.8%, *p* = 0.987) usage between the two groups. At a median follow-up of 36 months, DFS rates were 81.2% for the Hetero group and 96.5% for the Homo group (*p* = 0.041; adjusted *HR*, 2.95; 95% CI, 1.04–8.37). The estimated 3-year OS rates for the groups were 95.8% and 99.5%, respectively (*p* = 0.059; adjusted *HR*, 5.36; 95% CI, 0.97–29.69).

**Conclusion:**

Heterogeneity of ER, PR, HER2, or Ki67 was present in 24.0% patients with MMBC. Biomarkers heterogeneity influenced adjuvant endocrine therapy usage and was associated with worse disease outcomes, indicating further clinical evaluation.

## Introduction

Breast cancer is a heterogeneous group of diseases in which individual patient differs in morphological features, molecular profiles, therapeutic responses, and prognosis (1). Morphological variability such as pathological type and histological grade has been well documented for decades and forms the basis for histological classification of breast cancer. More recently, different molecular phenotypes of breast cancer have been defined by genetic or immunohistochemistry testing. For example, the well-defined 2013 St Gallen subtypes of breast cancer were based on the expressions of estrogen (ER) and progesterone (PR) receptors, human epidermal growth factor receptor 2 (HER2), and Ki67 proliferative index, which provide prognostic information and can be used to tailor systemic adjuvant therapy (2).

The molecular heterogeneity can occur either between different tumors within the same patient (intertumoral heterogeneity) or within the same tumor (intratumoral heterogeneity) (1). Heterogeneous expressions of ER, PR, HER2, and Ki67 have been widely reported between core needle biopsy and surgical samples, between different regions of a primary tumor, between a primary tumor and a matched metastatic lesion, or between metastatic lesions (3–9). Beyond spatial heterogeneity, heterogeneity can be observed as the natural evolution of a tumor or as consequences of anticancer treatments (10–12).

Multifocal/multicentric breast cancer (MMBC) has become more frequently diagnosed with the popular breast cancer screening program and the advancement of imaging methods (13, 14). In a previous study that evaluated the heterogeneity of ER, PR, HER2, and Ki67 between different foci in MMBC, the heterogeneity of these molecular markers was present in 4.4%, 15.9%, 9.7%, and 15.0% cases (13). MMBC with biomarkers heterogeneity represents a situation in breast cancer treatment where there are few guidelines to direct care. However, there are few studies investigating the therapeutic and prognostic impact of such heterogeneity. Herein, we performed this retrospective study to evaluate the rates of ER, PR, HER2, and Ki67 heterogeneity in patients with MMBC and its impacts on systemic adjuvant therapy decision-making and disease outcomes.

## Methods

### Study Population

Patients who received surgery and were diagnosed with multifocal or multicentric breast cancer at Department of General Surgery, Comprehensive Breast Health Center, Ruijin Hospital, Shanghai Jiao Tong University School of Medicine from January 2009 to December 2018 were retrospectively analyzed. Clinicopathological characteristics, adjuvant treatment, and follow-up data were retrieved from Shanghai Jiao Tong University Breast Cancer Database (SJTU-BCDB). The eligibility criteria were as follows: (1) at least one invasive tumor focus; (2) no distant metastasis at diagnosis; and (3) ER, PR, HER2, and Ki67 both tested between different tumor foci. Those who received neo-adjuvant therapy and those with only *in situ* tumor foci were excluded from the present study. Patients who did not have all samples tested for biomarkers were also exploratorily evaluated for disease outcomes.

### Histopathology Assessments

Histopathology analysis for different tumor foci on surgical specimens were independently performed and reviewed by two pathologists at the Department of Pathology, Ruijin Hospital, Shanghai Jiao Tong University School of Medicine (15, 16). In this study, multifocality was defined as the presence of more than one focus of carcinoma in one breast quadrant (MFBC), and multicentricity was defined as the presence of a focus in a different breast quadrant from the main lesion (MCBC) (13). Immunohistochemistry (IHC) of ER, PR, Ki67, and HER2 were performed on 4-µm slices of formalin-fixed paraffin-embedded (FFPE) specimens with primary antibodies against ER (SP1, 1:100, Dako, Denmark), PR (PgR 636, 1:100, Dako, Denmark), HER2 (4B5, Roche, Switzerland), Ki67 (MIB-1, 1:100, Dako, Denmark) by Ventana autostain system, BenchMark XT as previously described (15). In brief, the tissue sections were incubated with primary antibody of ER, PR, and Ki67 for 32 min at 42°C and of HER2 for 16 min at 42°C, which were then counterstained with hematoxylin. ER/PR was considered positive if there were ≥1% of the tumor cells with nuclear staining (16). HER2 was scored as 0 to 3+ by IHC, and those with IHC 2+ were further examined with fluorescence *in situ* hybridization (FISH) according to the ASCO/CAP guidelines, where HER2 positivity was defined as either IHC 3+ or IHC 2+ with FISH amplification (17–19). The Ki67 index was scored as the percentage of positively nuclear staining cells among at least 500–2,000 uniformly distributed cells or 2,000 cells from the hotspot and negative areas (20). Molecular subtypes were determined based on 2013 St Gallen system: luminal A-like (ER+/PR ≥ 20%/HER2-/Ki67 < 20%), luminal B-like (HER2−) (ER+/HER2−/Ki67 ≥ 20% or ER+/PR < 20%/HER2 or ER−/PR+/HER2−), luminal B-like (HER2+) (ER+ or PR+/HER2+), HER2+ (ER−/PR−/HER2+), and triple negative (ER−/PR−/HER2−) (2). Patients with concordant status of ER, PR, HER2, and Ki67 among all tumor foci were categorized into the homogeneous group (Homo group), while the heterogeneous group (Hetero group) was defined as the existence of at least one discordance for ER, PR, HER2, or Ki67 between different foci. The main focus referred to the largest tumor focus, and the other foci were named minor foci. Distance between the main and minor foci was assessed on pathological specimens, which was defined as the shortest distance between the edges of two tumor foci.

### Treatment and Follow-Up

Adjuvant treatment decisions were made through multidisciplinary team (MDT) meetings attended by surgical oncologists, medical oncologists, radiation oncologists, and pathologists (21). Decision was tailored according to the tumor biological features, stage at diagnosis, patient medical complications, and preferences. The patients were followed up every 3 months during the first 2 years after surgery, every 6 months from the third to the fifth year and once per year hereafter till death. DFS was defined as the period from the date of surgery to first local-regional relapse, contralateral breast cancer, secondary new malignant tumor, distant relapse, or death. OS was calculated from the date of surgery to the date of death. For patients who were free from DFS/OS events at the time of last follow-up, DFS/OS were calculated as the period from the date of surgery to the date of last follow-up.

### Statistics

Kappa tests were performed to evaluate the concordance rates of pathological type, histological grade, ER, PR, HER2, Ki67, and molecular subtype between the larger tumor focus and the smaller focus. For tumors with three or four foci, the results were considered concordant only when the biomarkers status of all tumor foci were concordant. The clinical features and adjuvant therapy options were compared between the Homo group and the Hetero group using chi-square test or Fisher’s exact test. DFS and OS rates were estimated from Kaplan–Meier curves and compared between the two groups *via* log-rank test. Cox proportional hazard model was used to calculate the hazard ratios for relapse and death. Clinical features and disease outcomes were also compared between MFBC and MCBC. Two-side *p* < 0.05 was considered statistically significant. All the statistical procedures were performed on SPSS (version 26.0).

## Results

### Baseline Clinicopathological Characteristics

**Figure 1:**
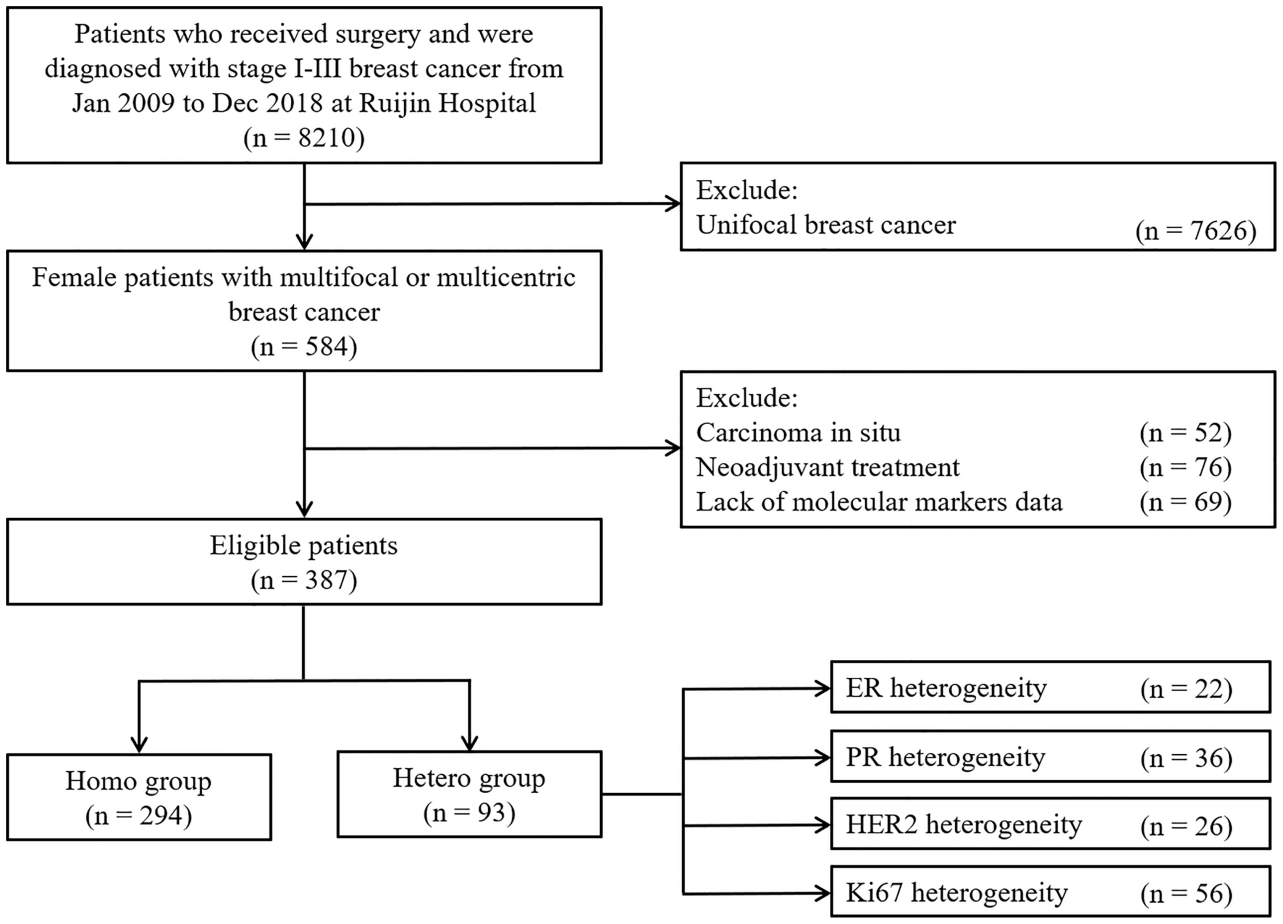
Flow chart of 387 patients in the study.

There were 8,210 stage II–III breast cancer patients who received surgery from January 2009 to December 2018 at Ruijin Hospital, among which 584 (7.11%) women were diagnosed with multifocal or multicentric breast cancers and 387 were included in the study (**Figure 1**). There were 52, 76, and 69 cases who were excluded from the study, as they had *in situ* foci only, received neo-adjuvant therapy, or lacked molecular markers data, respectively. Physical examination, sonography, mammography, and MRI identified 16.9%, 66.8%, 33.4%, and 77.2% of these patients, respectively (**Supplementary Figure S1**). As shown in the **Supplementary Figure S2**, the median distance between the main and minor foci was 12.6 [interquartile range (IQR), 7.2–20.0] mm, which showed no significant difference between the two groups (12.4 mm vs. 15.0 mm, *p* = 0.082).

The demographic and clinicopathological characteristics for the cohort are summarized in **Table 1**. The median age for the patients was 55 (IQR, 46–64) years, and 41.5% patients were pre/peri-menopausal at diagnosis. Patients with two foci accounted for 91.5% and 65.4% had multifocal diseases. Comparisons of MCBC and MFBC are summarized in **Supplementary Table S1**. Thirty-four (8.8%) patients received breast-conserving surgery, and sentinel lymph node biopsy was performed in 115 (31.3%) patients. There were 151 (39.0%) patients whose main tumor foci were larger than 2.0 cm, and 144 (37.2%) patients had positive axillary lymph nodes (ALN). A total of 77 (19.9%) and 140 (36.2%) were diagnosed with non-IDC in the main and minor tumor foci, respectively (**Supplementary Figure S3**). Luminal A-like, luminal B-like (HER2-), luminal B-like (HER2+), HER2+, and triple negative breast cancers were present in 117 (30.2%), 135 (34.9%), 50 (12.9%), 47 (12.1%), and 38 (9.9%) patients, respectively. There were significant differences in terms of pathological type of the minor tumor focus (*p* < 0.001) and molecular subtype of the main tumor focus (*p* < 0.001) between the Hetero group and the Homo group.

**Table 1:** Baseline clinical and pathological characteristics.

Characteristics	TotalN = 387 (%)	HomoN = 294 (%)	HeteroN = 93 (%)	*p*-value
**Age (y/o)**	**55 (46–64)**	**55 (46–64)**	**55 (47–65)**	**0.619**
**Menstrual status**				**0.538**
Pre/Peri-	160 (41.5)	124 (42.3)	36 (38.7)	
Post-	226 (58.5)	169 (57.7)	57 (51.3)	
**Number of foci**				**0.378**
2	354 (91.5)	271 (92.2)	83 (89.2)	
3/4	33 (8.5)	23 (7.8)	10 (10.8)	
**Location of foci**				**0.842**
Multifocal	253 (65.4)	193 (65.6)	60 (64.5)	
Multicentric	134 (34.6)	101 (34.4)	33 (35.5)	
**Breast surgery**				**0.727**
BCS	34 (8.8)	25 (8.5)	9 (9.7)	
Mastectomy	353 (91.2)	269 (91.5)	84 (90.3)	
**Axillary surgery**				**0.733**
SLNB	115 (31.3)	89 (30.9)	26 (32.9)	
ALND	252 (68.7)	199 (69.1)	53 (67.1)	
**Pathological type** ^a^				**0.656**
IDC	310 (80.1)	237 (80.6)	73 (78.5)	
Non-IDC	77 (19.9)	57 (19.4)	20 (21.5)	
**Pathological type** ^b^				**<0.001**
IDC	247 (63.8)	211 (71.8)	36 (38.7)	
Non-IDC	140 (36.2)	83 (28.2)	57 (61.3)	
**Tumor size** ^a^				**0.754**
≤2.0 cm	236 (61.0)	178 (60.5)	58 (62.4)	
>2.0 cm	151 (39.0)	116 (39.5)	35 (37.6)	
**ALN status**				**0.521**
Negative	243 (62.8)	182 (61.9)	61 (65.5)	
Positive	144 (37.2)	112 (38.1)	32 (34.4)	
**Histological grade** ^a^				**0.739**
I	24 (6.2)	20 (6.8)	4 (4.3)	
II	183 (47.3)	141 (48.0)	42 (45.2)	
III	96 (24.8)	71 (24.1)	25 (26.9)	
NA	84 (21.7)	62 (21.1)	22 (23.6)	
**Molecular subtype** ^a^				**<0.001**
LA	117 (30.2)	103 (35.0)	14 (15.1)	
LB (HER2−)	135 (34.9)	87 (29.6)	48 (51.6)	
LB (HER2+)	50 (12.9)	34 (11.6)	16 (17.2)	
HER2+	47 (12.1)	39 (13.3)	8 (8.6)	
TNBC	38 (9.9)	31 (10.5)	7 (7.5)	

a Main focus.

b Minor focus.

ALN, axillary lymph node; ALND, axillary lymph node dissection; BCS, breast-conserving surgery; HER2, human epidermal growth factor receptor 2; IDC, invasive ductal carcinoma; LA, Luminal A-like; LB, Luminal B-like; NA, not available; SLNB, sentinel lymph node biopsy; TNBC, triple negative breast cancer; y/o, years old.

### Rates of Molecular Markers Heterogeneity

As shown in **Table 2**, concordance rates of ER, PR, HER2, and Ki67 among different tumor foci were 94.3%, 90.7%, 93.3%, and 87.1%, respectively (all *p* values <0.001). Among the whole cohort, a total of 93 (24.0%) patients showed intertumoral heterogeneity of molecular markers, and the remaining 294 (76.0%) were homogeneous. There were 60 (23.7%) patients with MFBC and 33 (24.0%) with MCBC who were classified to the Hetero group (*p* = 0.842, **Supplementary Tables S1–3**).

**Table 2:** Concordance rates of pathological type, histological grade, ER, PR, HER2, and Ki67 status.

Main focus	Minor focus			Concordancerate (%)	Kappa	*p*-value
Pathological type	IDC	Non-IDC		78.0	0.473	<0.001
IDC	236	74				
Non-IDC	11	66				
Histological grade	I	II	III	88.4	0.772	<0.001
I	15	3	0			
II	3	139	5			
III	1	16	59			
ER	Negative	Positive		94.3	0.841	<0.001
Negative	79	8				
Positive	14	286				
PR	Negative	Positive		90.7	0.798	<0.001
Negative	121	18				
Positive	18	230				
HER2	Negative	Positive		93.3	0.819	<0.001
Negative	279	11				
Positive	15	82				
Ki67	< 20%	≥ 20%		87.1	0.743	<0.001
< 20%	163	8				
≥ 20%	42	174				

ER, estrogen receptor; HER2, human epidermal growth factor receptor 2; PR, progesterone receptor.

The molecular subtypes were identical within the same patient in 310 (80.1%) of the 387 cases using the 2013 St Gallen standard, with 104 luminal A-like tumors, 93 luminal B-like (HER2−) tumors, 42 luminal B-like (HER2+) tumors, 39 HER2-enriched tumors, and 32 triple negative breast cancers (**Table 3**). Molecular subtypes differed among different tumor foci in 77 (19.9%) patients, including 46 (18.2%) and 31 (23.1%) with MFBC and MCBC, respectively (*p* = 0.336, **Supplementary Tables S1, 4, 5**).

**Table 3:** Concordance rates of molecular subtypes^a^.

Main focus	Minor focus					Concordance rate (%)	Kappa	*p*-value
	LA	LB (HER2−)	LB (HER2+)	HER2+	TNBC	80.1	0.830	<0.001
LA	104	6	1	3	3			
LB (HER2−)	32	93	4	1	5			
LB (HER2+)	4	4	42	0	0			
HER2+	0	0	2	39	6			
TNBC	3	1	1	1	32			

a The cutoff value of Ki67 was 20% for differentiating luminal A-like and luminal B-like (HER2−).

HER2, human epidermal growth factor receptor 2; HR, hormone receptor; LA, luminal A-like; LB, luminal B-like; TNBC, triple negative breast cancer.

### Heterogeneity of Molecular Markers and Adjuvant Therapy

**Figure 2:**
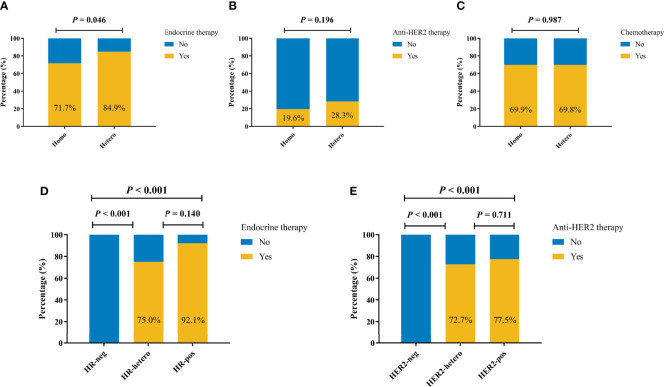
Adjuvant systemic therapy by molecular markers status among 330 patients with at least two invasive tumor foci. Adjuvant endocrine therapy **(A)**, anti-HER2 therapy **(B)**, and chemotherapy **(C)** by molecular markers status. **(D)** Adjuvant endocrine therapy by HR status. **(E)** Adjuvant anti-HER2 therapy by HER2 status.

There were 150 patients with recorded MDT-recommended adjuvant therapies, and the compliance was 95.3%, 96.0%, and 97.3% to chemotherapy, endocrine therapy, and anti-HER2 therapy, respectively. A total of 45 (84.9%) out of 330 patients with at least two invasive tumor foci in the Hetero group received adjuvant endocrine therapy, which was significantly higher than that of patients in the Homo group (71.7%, *p* = 0.046, **Figure 2A**). There were no significant differences in the usage rates of adjuvant anti-HER2 therapy (28.3% vs. 19.6%, *p* = 0.196, **Figure 2B**) and chemotherapy (69.9% vs. 69.8%, *p* = 0.987, **Figure 2C**) between the two groups.

As shown in **Figure 2D**, endocrine therapy was more frequently utilized among patients with HR heterogeneity than HR-negative patients (75.0% vs. 0.0%, *p* < 0.001), while the rates were comparable among patients with at least one HR+ tumor foci (75.0% vs. 92.1%, *p* = 0.140). Similarly, HER2 heterogeneity was associated with higher rate of anti-HER2 therapy compared with HER2-negative patients (72.7% vs. 0.0%, *p* < 0.001), and once again, no significant difference was observed among patients with at least one HER2+ tumor focus (72.7% vs. 77.5%, *p* = 0.711, **Figure 2E**).

### Heterogeneity of Molecular Markers and Disease Outcomes

**Figure 3:**
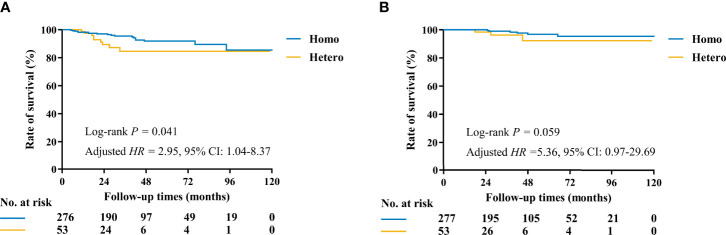
Kaplan–Meier curves of DFS and OS by molecular markers status among 330 patients with at least two invasive tumor foci. **(A)** The estimated 3-year DFS rates for the Homo and Hetero groups were 96.5% and 81.2%, respectively (*p* = 0.041). **(B)** The estimated 3-year OS rates for the Homo and Hetero groups were 99.5% and 95.8%, respectively (*p* = 0.059).

At a median follow-up of 35 (IQR, 19–57) months, 21 DFS events and 5 deaths were recorded (**Table 4**). Patients in the Hetero group had significantly worse DFS (81.2% vs. 96.5%, *p* = 0.041) and comparable OS (95.8% vs. 99.5%, *p* = 0.059) than those in the Homo group (**Table 5** and **Figure 3**). After adjusting age, tumor size, ALN status, molecular subtype, and systemic treatments in multivariate models, patients in the Hetero group had significantly worse DFS (adjusted *HR*, 2.95; 95% CI, 1.04–8.37) and comparable OS (adjusted *HR*, 5.36; 95% CI, 0.97–29.69) than those in the Homo group (**Supplementary Table S1**).

**Table 4:** Details of DFS and OS events by status of molecular markers among 330 patients with at least two invasive tumor foci.

	TotalN = 330 (%)	HomoN = 277 (%)	HeteroN = 53 (%)
**DFS events**			
No recurrence	309 (93.6)	261 (94.2)	48 (90.6)
Local-regional recurrence	5 (1.5)	4 (1.4)	1 (1.9)
Contralateral breast cancer	4 (1.2)	4 (1.4)	0 (0.0)
Second non-breast malignancy	2 (0.6)	1 (0.4)	1 (1.9)
Distant recurrence	8 (2.4)	6 (2.2)	2 (3.8)
Death without recurrence	2 (0.6)	1 (0.4)	1 (1.9)
**OS events**			
Alive	323 (97.9)	272 (98.2)	51 (96.2)
Death of any cause	7 (2.1)	5 (1.8)	2 (3.9)
Death with recurrence	5 (1.5)	4 (1.4)	1 (1.9)
Death without recurrence	2 (0.6)	1 (0.4)	1 (1.9)

DFS, disease-free survival; OS, overall survival.

**Table 5:** Univariate analysis of prognostic factors affecting DFS and OS among 330 patients with at least two invasive tumor foci.

Characteristics	*p*-value	
	DFS	OS
Age (<50 y/o vs. ≥50 y/o)	0.741	0.277
Menstrual status (Pre/Peri- vs. Post-)	0.966	0.969
Number of foci (2 vs. 3/4)	0.264	0.557
Location of foci (Multifocal vs. Multicentric)	0.571	0.703
Histology type (IDC vs. non-IDC) ^a^	0.802	0.789
Tumor size (≤2.0 vs. >2.0 cm) ^a^	0.786	0.292
ALN status (Negative vs. Positive)	0.572	0.841
Histological grade (I vs. II vs. III vs. NA)^a^	0.637	0.892
Molecular subtype^a^	0.309	0.460
Chemotherapy (No vs. Yes)	0.698	1.000
Endocrine therapy (No vs. Yes)	0.102	0.458
Anti-HER2 therapy (No vs. Yes)	0.379	0.352
Group (Homo vs. Hetero)	0.041	0.059

a Main focus.

ALN, axillary lymph node; IDC, invasive ductal carcinoma; DFS, disease-free survival; NA, not available; OS, overall survival; y/o, years old.

## Discussion

The study was designed to evaluate the rates of molecular markers heterogeneity and its associations with systemic adjuvant therapy and disease outcomes in MMBC. Molecular markers showed good concordance among different tumor foci. Heterogeneity of ER, PR, HER2, and Ki67 were present in 24.0% MMBC, which was associated with more adjuvant endocrine therapy usage (*p* = 0.046) and shorter DFS (*p* = 0.041), indicating the necessity of molecular assessments for different tumor foci in patients with MMBC.

There were some published literatures that reported the rates of intertumoral biomarkers heterogeneity among different foci in MMBC (13, 22–25). For example, Buggi and colleagues enrolled 113 invasive multiple breast cancers, and they reported mismatches on ER, PR, HER2, and Ki67 in 4.4%, 15.9%, 9.7%, and 15.0% cases (13). Similarly, the rate of ER, PR, HER2, and Ki67 heterogeneity in our cohort was 5.7%, 9.3%, 6.7%, and 22.9%, respectively. Moreover, molecular subtypes differed in 77 (19.9%) patients as classified by 2013 St Gallen system, comparable to the results of Pekar et al. (12.7%) (14). There might be some causes of molecular heterogeneity. First, heterogeneity of molecular markers was more frequent among patients whose minor tumor focus was invasive carcinoma of special type or carcinoma *in situ* in our study, making pathological differences a potential explanation. This finding will guide us to select patients for a second molecular evaluation in the present clinical practice. However, there would be other innate tumor properties beyond histopathology but crucial to tumor tumorigenesis and evolution, which control tumor heterogeneity. Recent studies have revealed that extensive genetic diversity caused by genome instability and mutation will affect key cancer pathways, eventually driving phenotypic variation (10–12, 26). In light of this, intratumor heterogeneity can lead to underestimation of the tumor genomics landscape from pathology only and may present major challenges to personalized medicine, which should be further evaluated by emerging technologies such as next-generation and single-cell sequencing (12). Moreover, non-mutational epigenetic reprogramming and cellular plasticity can also contribute to tumor heterogeneity (27, 28). Last but not the least, technical issue and analytical artifact may also affect accuracy of molecular evaluation, which could be avoided by standardization.

Accurate biomolecular analysis is of great significance to make treatment options and to evaluate functional outcomes and quality of life of breast cancer patients in the era of precise medicine (2, 29–31). However, with limited knowledge on the consequences of molecular heterogeneity for therapeutic decision-making, it has been accepted that biomarkers can be assessed only in the largest individual tumor focus (32). This is based on the observations that molecular markers in MMBC are usually homogeneous. However, if the tumor foci demonstrate different pathological or histological features, biomarkers evaluation of the smaller focus will be necessary. According to Buggi et al., 14 out of the 113 (12.4%) patients received additional systemic treatments with the biomarkers analysis for the smaller tumor focus (13). In the present study, heterogeneity of biomarkers was also found to be significantly associated with the usage of adjuvant endocrine therapy among patients with at least two invasive tumor foci. Additional biomarkers evaluation of the other foci would potentially change the adjuvant treatment decisions, especially for those whose main tumor focus lacked HR or HER2. This would further impact breast cancer patient’s survival.

To the best of the authors’ knowledge, this was the largest study to evaluate the prognostic significance of intertumoral biomarkers heterogeneity. Our cohort revealed that patients in the Hetero group had clinically worse DFS and OS compared those in the Homo group, although the differences were not statistically significant. However, the results should be taken as exploratory only and interpreted with caution, particularly given the relatively small number of DFS and OS events and short follow-up. Consistent with our results, Pekar et al. concluded that patients with phenotypically heterogeneous MMBC had a significantly shorter breast-cancer-specific survival (*HR* = 2.87; 95% CI, 1.08–7.64, *p* = 0.034) and OS (*HR* = 2.80; 95% CI, 1.05–7.44, *p* = 0.039) (14). The inferior disease outcomes in the Hetero group were thought to be associated with the biology behavior itself and possible undertreatment or low treatment sensitivity. Taken together, these results suggested the necessity of evaluating molecular markers for different tumor foci in patients with MMBC (33). However, we acknowledge that it is not a routine to test all tumor foci in the present clinical practice. Possibilities could be attributed to lack of evidence or economic reasons. In addition, it is important to point out that patients who have MMBC but do not have all foci tested may be more likely to have homogeneous tumors, since the finding of MMBC with discordant grades may prompt biomarker evaluation. Therefore, it could be useful to evaluate the clinical features and disease outcomes among patients who do not have all samples tested for biomarkers. However, this was a small group in our cohort, and only 3 out of 30 patients with multiple diseases who lacked biomarkers data experienced DFS events, indicating that a large cohort with more patients are needed to validate this recommendation.

The present study enrolled both multifocal and multicentric diseases, which were heterogeneous and could be further differentiated into MFBC and MFBC. Patients with MCBC were different to those with MFBC in terms of number of tumor foci, tumor size, and molecular subtype (**Supplementary Table S1**). However, the biomarker and subtype heterogeneity rates were comparable between the two subgroups (**Supplementary Table S1–S5**), while for DFS and OS, no significant differences were observed between the two groups (**Supplementary Figure S3**). To date, relatively few reports have directly compared the clinical–pathological features, treatment patterns, and survivals of patients with MFBC and MCBC, which warrant further research (34–36).

The incidence of MMBC increases with the advancement of preoperative imaging, and intertumoral molecular heterogeneity has attracted the attention of clinicians. For example, MRI can identify 74.6%, 54.2%, and 67.3% of MMBC that were not identified by physical examination, sonography, or mammography, respectively (**Supplementary Figure S1**). We performed the study for the first time to evaluate the rates of biomarkers heterogeneity in MMBC and its impacts on adjuvant therapy and survival. However, there were several limitations in the present study. First, this was a single-institutional retrospective study, so there might be selection bias and limited applicability. Further validation in other cohorts will provide us more insights to the therapeutic and prognostic role of biomarkers heterogeneity. Second, the number of DFS and OS events was very small, and survival did not differ significantly between the groups. Therefore, the evaluation of outcomes was exploratory and hypothesis generating and warranted larger study. Third, the present study enrolled patients over a 9-year period from January 2009 through December 2018, which might exert an influence to disease outcomes.

In conclusion, heterogeneity of ER, PR, HER2, or Ki67 was present in 24.0% patients with MMBC. Biomarkers heterogeneity was associated with more adjuvant endocrine therapy usage and worse disease outcomes, indicating the necessity of molecular assessments for different tumor foci in patients with MMBC.

## Data Availability Statement

The raw data supporting the conclusions of this article will be made available by the authors, without undue reservation.

## Author Contributions

XC conceived and designed the study. SL analyzed and interpreted the data for presentation and was a main contributor in writing the manuscript. XC revised the manuscript. All authors contributed to the article and approved the submitted version.

## Funding

This study was funded by the National Natural Science Foundation of China (Grant Number: 81772797); Shanghai Municipal Education Commission—Gaofeng Clinical Medicine Grant Support (20172007); and Ruijin Hospital, Shanghai Jiao Tong University School of Medicine-”Guangci Excellent Youth Training Program” (GCQN-2017-A18). All these financial sponsors had no role in the study design, data collection, analysis, or interpretation.

## Conflict of Interest

The authors declare that the research was conducted in the absence of any commercial or financial relationships that could be construed as a potential conflict of interest.

## Publisher’s Note

All claims expressed in this article are solely those of the authors and do not necessarily represent those of their affiliated organizations, or those of the publisher, the editors and the reviewers. Any product that may be evaluated in this article, or claim that may be made by its manufacturer, is not guaranteed or endorsed by the publisher.
