# Supplementary material for: Association of Molecular Biomarker Heterogeneity With Treatment Pattern and Disease Outcomes in Multifocal or Multicentric Breast Cancer
Source: Front Oncol. 2022 Jun 23;12:833093. doi: 10.3389/fonc.2022.833093 (PMC9259989; doi:10.3389/fonc.2022.833093)
Supplement: Supplementary file 1 [file DataSheet_1.docx]

**Association of molecular biomarkers heterogeneity and treatment pattern, disease outcomes in multifocal or multicentric breast cancer patients**

**Authors:**

Shuai Li ^1^, Jiayi Wu ^1^, Ou Huang ^1^, Jianrong He ^1^, Weiguo Chen ^1^, Yafen Li ^1^, Xiaosong Chen ^1^*, Kunwei Shen ^1^*

**Affiliation:**

^1^ Department of General Surgery, Comprehensive Breast Health Center, Ruijin Hospital, Shanghai Jiao Tong University School of Medicine, Shanghai 200025, China

**Correspondence to:**

Xiaosong Chen, 22nd Floor, 197 Ruijin Er Road, Shanghai 200025, China. E-mail: chenxiaosong0156@hotmail.com

Kunwei Shen, 22nd Floor, 197 Ruijin Er Road, Shanghai 200025, China. E-mail: kwshen@medmail.com.cn

**Supplementary Table S1** Baseline clinical and pathological characteristics by location of tumor foci

| Characteristics | Total  N = 387 (%) | MFBC  N = 253 (%) | MCBC  N = 134 (%) | *P* value |
| --- | --- | --- | --- | --- |
| **Age (y/o)** | **55 (46-64)** | **55 (46-63)** | **55 (46-65)** | **0.647** |
| **Menstrual status** |  |  |  | **0.581** |
| Pre/Peri- | 160 (41.5) | 107 (42.5) | 53 (39.6) |  |
| Post- | 226 (58.5) | 145 (57.5) | 81 (60.4) |  |
| **Number of foci** |  |  |  | **< 0.001** |
| 2 | 354 (91.5) | 241 (95.3) | 113 (84.3) |  |
| 3/4 | 33 (8.5) | 12 (4.7) | 21 (15.7) |  |
| **Breast surgery** |  |  |  | **< 0.001** |
| BCS | 34 (8.8) | 34 (13.4) | 0 (0.0) |  |
| Mastectomy | 353 (91.2) | 219 (86.6) | 134 (100.0) |  |
| **Axillary surgery** |  |  |  | **0.218** |
| SLNB | 115 (31.3) | 70 (29.2) | 45 (35.4) |  |
| ALND | 252 (68.7) | 170 (70.8) | 82 (64.6) |  |
| **Pathological type ^a^** |  |  |  | **0.212** |
| IDC | 310 (80.1) | 198 (78.3) | 112 (83.6) |  |
| Non-IDC | 77 (19.9) | 55 (21.7) | 22 (16.4) |  |
| **Pathological type ^b^** |  |  |  | **0.440** |
| IDC | 247 (63.8) | 158 (62.5) | 89 (66.4) |  |
| Non-IDC | 140 (36.2) | 95 (37.5) | 45 (33.6) |  |
| **Tumor size ^a^** |  |  |  | **0.019** |
| ≤ 2.0 cm | 236 (61.0) | 165 (65.2) | 71 (53.0) |  |
| > 2.0 cm | 151 (39.0) | 88 (34.8) | 63 (47.0) |  |
| **ALN status** |  |  |  | **0.072** |
| Negative | 243 (62.8) | 167 (66.0) | 76 (56.7) |  |
| Positive | 144 (37.2) | 86 (34.0) | 58 (43.3) |  |
| **Histological grade ^a^** |  |  |  | **0.568** |
| Ⅰ | 24 (6.2) | 13 (5.1) | 11 (8.2) |  |
| Ⅱ | 183 (47.3) | 121 (47.8) | 62 (46.3) |  |
| Ⅲ | 96 (24.8) | 61 (24.1) | 35 (26.1) |  |
| NA | 84 (21.7) | 58 (23.0) | 26 (19.4) |  |
| **Histological grade ^b^** |  |  |  | **0.445** |
| Ⅰ | 19 (4.9) | 11 (4.3) | 8 (6.0) |  |
| Ⅱ | 161 (41.6) | 100 (39.5) | 61 (45.5) |  |
| Ⅲ | 63 (16.3) | 41 (16.2) | 22 (16.4) |  |
| NA | 144 (37.2) | 101 (40.0) | 43 (32.1) |  |
| **Molecular subtype ^a^** |  |  |  | **0.013** |
| LA | 117 (30.2) | 69 (27.3) | 48 (35.8) |  |
| LB (HER2-) | 135 (34.9) | 81 (32.0) | 54 (40.3) |  |
| LB (HER2+) | 50 (12.9) | 35 (13.8) | 15 (11.2) |  |
| HER2+ | 47 (12.1) | 39 (15.4) | 8 (6.0) |  |
| TNBC | 38 (9.9) | 29 (11.5) | 9 (6.7) |  |
| **Molecular subtype ^b^** |  |  |  | **0.053** |
| LA | 143 (37.0) | 85 (33.7) | 58 (43.3) |  |
| LB (HER2-) | 104 (26.9) | 63 (24.9) | 41 (30.6) |  |
| LB (HER2+) | 50 (12.9) | 36 (14.2) | 14 (10.4) |  |
| HER2+ | 44 (11.4) | 34 (13.4) | 10 (7.5) |  |
| TNBC | 46 (11.8) | 35 (13.8) | 11 (8.2) |  |
| **Molecular heterogeneity** |  |  |  | **0.842** |
| No | 294 (76.0) | 193 (76.3) | 101 (75.4) |  |
| Yes | 93 (24.0) | 60 (23.7) | 33 (24.0) |  |
| **Subtype heterogeneity** |  |  |  | **0.336** |
| No | 310 (80.1) | 207 (81.8) | 103 (76.9) |  |
| Yes | 77 (19.9) | 46 (18.2) | 31 (23.1) |  |

**^a^** : main focus; **^b^** : minor focus.

Abbreviations: ALN, axillary lymph node; ALND: axillary lymph node dissection; BCS, breast-conserving surgery; HER2, human epidermal growth factor receptor 2; IDC: invasive ductal carcinoma; LA, Luminal A-like; LB, Luminal B-like; NA: not available; SLNB: sentinel lymph node biopsy; TNBC, triple negative breast cancer; y/o, years old.

**Supplementary Table S2** Concordance rates of pathological type, histological grade, ER, PR, HER2, and Ki67 status in multifocal breast cancer

| Main focus | Minor focus | | | | Concordance  rate (%) | Kappa | *P* value |
| --- | --- | --- | --- | --- | --- | --- | --- |
| Pathological type | IDC | | Non-IDC | | 76.7 | 0.503 | < 0.001 |
| IDC | 151 | | 47 | |  |  |  |
| Non-IDC | 7 | | 48 | |  |  |  |
| Histological grade | Ⅰ | Ⅱ | | Ⅲ | 90.6 | 0.812 | < 0.001 |
| Ⅰ | 10 | 1 | | 0 |  |  |  |
| Ⅱ | 1 | 89 | | 4 |  |  |  |
| Ⅲ | 0 | 8 | | 36 |  |  |  |
| ER | Negative | | Positive | | 94.5 | 0.862 | < 0.001 |
| Negative | 63 | | 6 | |  |  |  |
| Positive | 8 | | 176 | |  |  |  |
| PR | Negative | | Positive | | 92.5 | 0.846 | < 0.001 |
| Negative | 98 | | 12 | |  |  |  |
| Positive | 7 | | 136 | |  |  |  |
| HER2 | Negative | | Positive | | 92.5 | 0.815 | < 0.001 |
| Negative | 172 | | 7 | |  |  |  |
| Positive | 12 | | 62 | |  |  |  |
| Ki67 | < 20% | | ≥ 20% | | 88.1 | 0.763 | < 0.001 |
| < 20% | 104 | | 5 | |  |  |  |
| ≥ 20% | 25 | | 119 | |  |  |  |

Abbreviations: ER: estrogen receptor; HER2, human epidermal growth factor receptor 2; PR: progesterone receptor.

**Supplementary Table S3** Concordance rates of pathological type, histological grade, ER, PR, HER2, and Ki67 status in multicentric breast cancer

| Main focus | Minor focus | | | | Concordance  rate (%) | Kappa | *P* value |
| --- | --- | --- | --- | --- | --- | --- | --- |
| Pathological type | IDC | | Non-IDC | | 76.8 | 0.406 | < 0.001 |
| IDC | 85 | | 27 | |  |  |  |
| Non-IDC | 4 | | 18 | |  |  |  |
| Histological grade | Ⅰ | Ⅱ | | Ⅲ | 81.9 | 0.660 | < 0.001 |
| Ⅰ | 5 | 2 | | 0 |  |  |  |
| Ⅱ | 2 | 46 | | 1 |  |  |  |
| Ⅲ | 1 | 10 | | 21 |  |  |  |
| ER | Negative | | Positive | | 94.0 | 0.765 | < 0.001 |
| Negative | 16 | | 2 | |  |  |  |
| Positive | 6 | | 110 | |  |  |  |
| PR | Negative | | Positive | | 87.3 | 0.648 | < 0.001 |
| Negative | 23 | | 6 | |  |  |  |
| Positive | 11 | | 100 | |  |  |  |
| HER2 | Negative | | Positive | | 94.8 | 0.819 | < 0.001 |
| Negative | 107 | | 4 | |  |  |  |
| Positive | 3 | | 20 | |  |  |  |
| Ki67 | < 20% | | ≥ 20% | | 85.0 | 0.704 | < 0.001 |
| < 20% | 59 | | 3 | |  |  |  |
| ≥ 20% | 17 | | 55 | |  |  |  |

Abbreviations: ER: estrogen receptor; HER2, human epidermal growth factor receptor 2; PR: progesterone receptor.

**Supplementary Table S4** Concordance rates of molecular subtypes ^a^ in multifocal breast cancer

| Main focus | Minor focus | | | | | Concordance rate (%) | Kappa | *P* value |
| --- | --- | --- | --- | --- | --- | --- | --- | --- |
|  | LA | LB (HER2-) | LB (HER2+) | HER2+ | TNBC | 81.8 | 0.765 | < 0.001 |
| LA | 64 | 2 | 0 | 1 | 2 |  |  |  |
| LB (HER2-) | 18 | 57 | 3 | 1 | 2 |  |  |  |
| LB (HER2+) | 2 | 3 | 30 | 0 | 0 |  |  |  |
| HER2+ | 0 | 0 | 2 | 31 | 6 |  |  |  |
| TNBC | 1 | 1 | 1 | 1 | 25 |  |  |  |

^a^ : The cut-off value of Ki67 was 20% for differentiating Luminal A-like and Luminal B-like (HER2-).

Abbreviations: HER2, human epidermal growth factor receptor 2; HR, hormone receptor; LA, Luminal A-like; LB, Luminal B-like; TNBC, triple negative breast cancer.

**Supplementary Table S5** Concordance rates of molecular subtypes ^a^ in multicentric breast cancer

| Main focus | Minor focus | | | | | Concordance rate (%) | Kappa | *P* value |
| --- | --- | --- | --- | --- | --- | --- | --- | --- |
|  | LA | LB (HER2-) | LB (HER2+) | HER2+ | TNBC | 76.9 | 0.670 | < 0.001 |
| LA | 40 | 4 | 1 | 2 | 1 |  |  |  |
| LB (HER2-) | 14 | 36 | 1 | 0 | 3 |  |  |  |
| LB (HER2+) | 2 | 1 | 12 | 0 | 0 |  |  |  |
| HER2+ | 0 | 0 | 0 | 8 | 0 |  |  |  |
| TNBC | 2 | 0 | 0 | 0 | 7 |  |  |  |

^a^ : The cut-off value of Ki67 was 20% for differentiating Luminal A-like and Luminal B-like (HER2-).

Abbreviations: HER2, human epidermal growth factor receptor 2; HR, hormone receptor; LA, Luminal A-like; LB, Luminal B-like; TNBC, triple negative breast cancer.

**Supplementary Table S6** Multivariate analysis of prognostic factors associated with DFS and OS.

| Characteristics | DFS | |  | OS | |
| --- | --- | --- | --- | --- | --- |
|  | HR (95% CI) | *P* value |  | HR (95% CI) | *P* value |
| **Group** |  | **0.042** |  |  | **0.055** |
| Homo | 1.00 |  |  | 1.00 |  |
| Hetero | 2.95 (1.04-8.37) |  |  | 5.36 (0.97-29.69) |  |
| **Age (y/o)** |  | **0.752** |  |  | **0.352** |
| ≤ 50 | 1.00 |  |  | 1.00 |  |
| > 50 | 0.93 (0.36-2.43) |  |  | 0.45 (0.08-2.41) |  |
| **Tumor size ^a^** |  | **0.987** |  |  | **0.250** |
| ≤ 2.0 cm | 1.00 |  |  | 1.00 |  |
| > 2.0 cm | 0.95 (0.36-2.45) |  |  | 2.76 (0.49-15.54) |  |
| **ALN status** |  | **0.707** |  |  | **0.806** |
| Negative | 1.00 |  |  | 1.00 |  |
| Positive | 1.20 (0.47-3.09) |  |  | 0.78 (0.11-5.58) |  |
| **Molecular subtype ^a^** |  | **0.561** |  |  | **0.881** |
| LA | 1.00 |  |  | 1.00 |  |
| LB (HER2-) | 3.01 (0.80-11.37) |  |  | 3.38 (0.31-36.34) |  |
| LB (HER2+) | 2.26 (0.45-11.37) |  |  | 2.13 (0.11-42.55) |  |
| HER2+ | 2.24 (0.37-13.45) |  |  | 3.62 (0.19-70.90) |  |
| TNBC | 1.19 (0.12-11.49) |  |  | ∞ (∞-∞) |  |
| **Endocrine therapy** |  | **0.917** |  |  | **0.971** |
| No | 1.00 |  |  | 1.00 |  |
| Yes | ∞ (∞-∞) |  |  | ∞ (∞-∞) |  |
| **Anti-HER2 therapy** |  | **0.343** |  |  | **0.206** |
| No | 1.00 |  |  | 1.00 |  |
| Yes | 3.23 (0.29-36.43) |  |  | 2.52 (0.60-10.59) |  |
| **Chemotherapy** |  | **0.987** |  |  | **0.789** |
| No | 1.00 |  |  | 1.00 |  |
| Yes | 1.01 (0.29-3.52) |  |  | 0.78 (0.13-4.71) |  |

**^a^** : main focus.

Abbreviations: ALN, axillary lymph node; LA, Luminal A-like; LB, Luminal B-like; TNBC, triple negative breast cancer; y/o, years old.


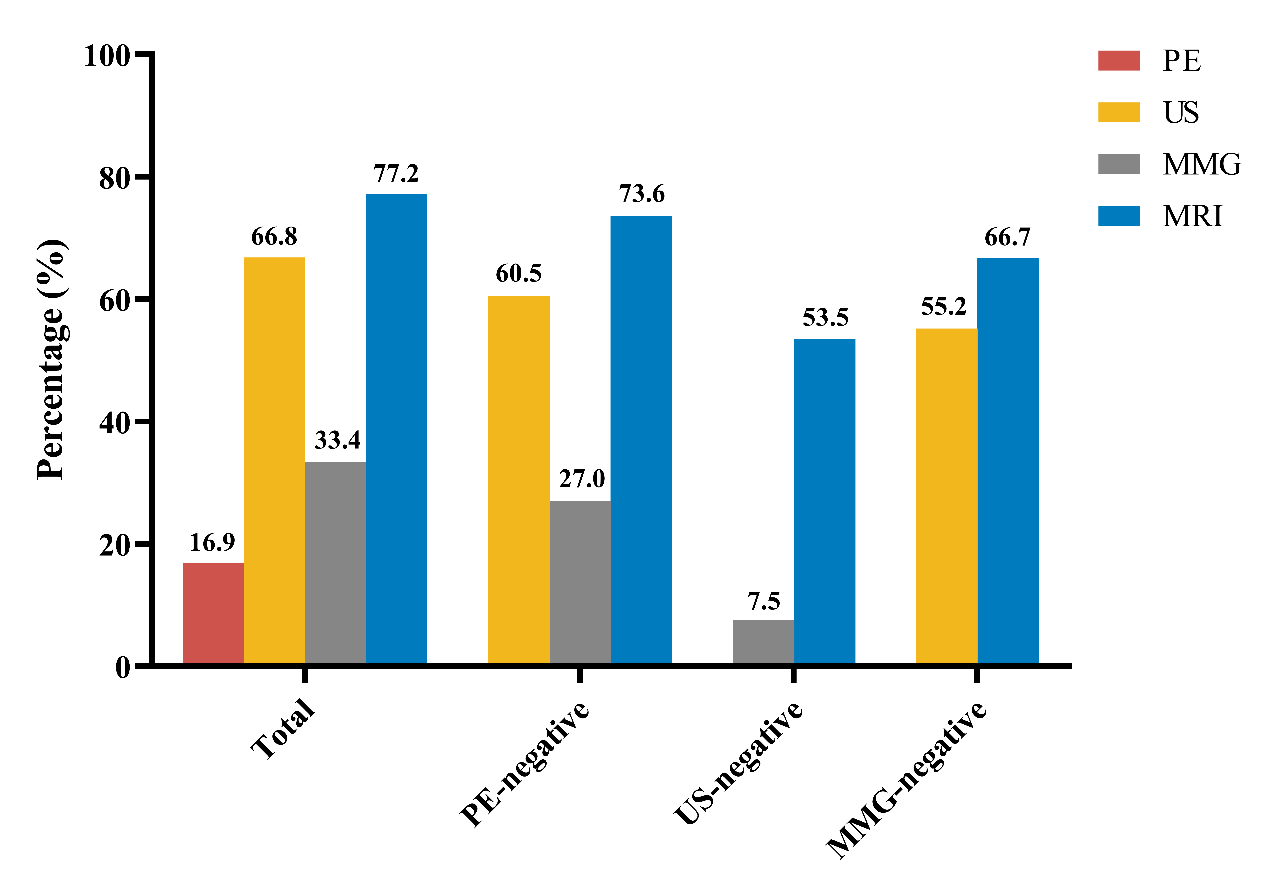


**Supplementary Figure 1** Percentages of patients with MMBC that could be identified by physical examination, ultrasound, mammography, and MRI.

Abbreviation: PE, physical examination; US: ultrasound; MMG: mammography; MRI: magnetic resonance imaging





**Supplementary Figure S2** Scatter diagram of distance between major and minor foci in the whole cohort and the Homo, Hetero groups.





**Supplementary Figure S3** Details of non-IDC pathological types of the main focus (A) and minor focus (B).


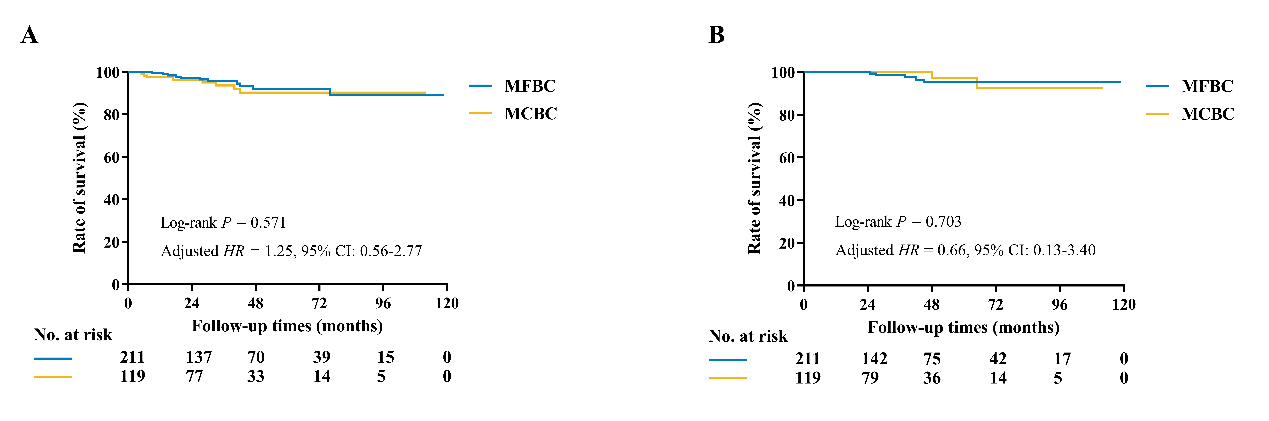


**Supplementary Figure S4** Kaplan-Meier curves of DFS and OS by location of tumor foci among 330 patients with at least 2 invasive tumor foci. (A) The estimated 3-year DFS rates for the MFBC and MCBC groups were 93.8% and 92.5%, respectively (*P* = 0.571). (B) The estimated 3-year OS rates for the MFBC and MCBC groups were 97.7% and 100.0%, respectively (*P* = 0.703).
